# Neutrophil recruitment depends on platelet-derived leukotriene B4

**DOI:** 10.1182/bloodadvances.2024014947

**Published:** 2025-03-10

**Authors:** Christian Hackenbroch, Tugce Cimen, Carina Gross, Stefanie Rubenzucker, Philipp Burkard, Niklas Groß, Robert Ahrends, Tamara Girbl, David Stegner

**Affiliations:** 1 Julius-Maximilians University of Würzburg, Rudolf Virchow Center for Integrative and Translational Bioimaging, Würzburg, Germany; 2 University Hospital Würzburg, Institute of Experimental Biomedicine I, Würzburg, Germany; 3 Department of Analytical Chemistry, University of Vienna, Vienna, Austria; 4 Vienna Doctoral School in Chemistry, University of Vienna, Vienna, Austria

**Figure:**
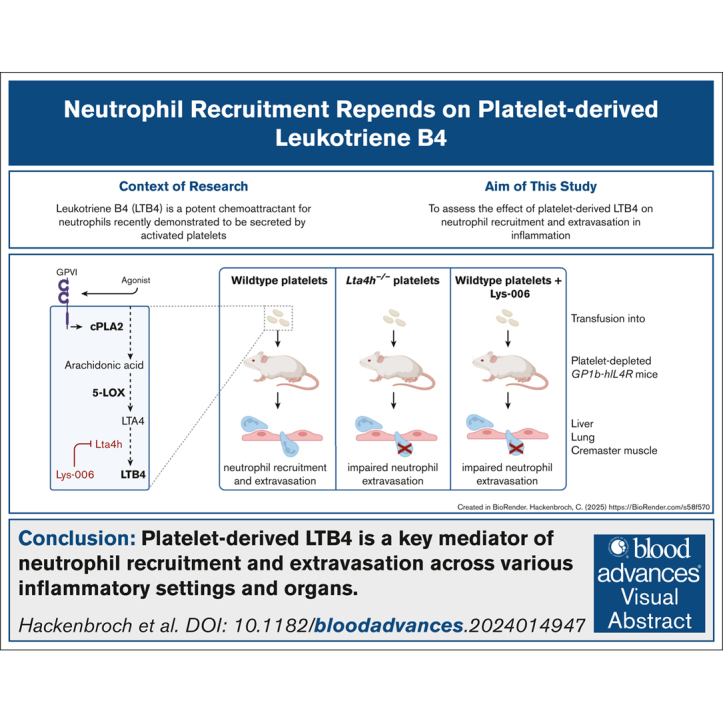

**TO THE EDITOR:**

Neutrophils, the most abundant leukocytes, are crucial in the immune response against infections.^1^ However, during sterile inflammation, their activity can be harmful. They release proteases, reactive oxygen species, and cytokines, thereby exacerbating inflammation, and this can lead to chronic inflammation and tissue damage.^2^ Leukotriene B4 (LTB4), a potent lipid mediator secreted by neutrophils, acts as a chemoattractant that amplifies neutrophil recruitment.^3^ LTB4 is synthesized from arachidonic acid, which is cleaved from cellular membranes by cytosolic phospholipase A2 and processed through the 5-lipoxygenase (5-LOX) pathway to form leukotriene A4 (LTA4). LTA4 is then converted to bioactive LTB4 by LTA4 hydrolase (Lta4h).^4^ Released via extracellular vesicles,^5^ LTB4 signals through the leukotriene B4 receptor 1 (BLT1) receptor to drive neutrophil recruitment and extravasation and to enhance effector functions like chemokine release and reactive oxygen species production.^4^^,^^5^ Elevated LTB4 levels are linked to acute and chronic inflammatory diseases, including autoimmune and allergic conditions.^4^ Targeting the LTB4-BLT1 axis has been explored pharmacologically. The 5-LOX inhibitor zileuton is approved for clinical use, but it affects additional 5-LOX–dependent mediators besides LTB4. Consequently, Lta4h has emerged as a promising, more specific target, and new inhibitors, like Lys-006, are currently in phase 2 trials for conditions such as nonalcoholic fatty liver disease and inflammatory acne.^6^^,^^7^

Recent lipidomic studies have revealed that platelets, traditionally known for their role in hemostasis, are also capable of generating LTB4.^8^ Although platelets are known contributors to inflammation,^9^ the role of platelet-derived LTB4 remains unclear. In this study, we investigated its contribution to neutrophil recruitment and extravasation using Lta4h-deficient mice (*Lta4h*^*–/–*^) and Lys-006–treated platelets, demonstrating the essential role of platelet-derived LTB4 in modulating neutrophil responses in inflammation.

To specifically investigate the role of platelet-derived LTB4, we used *Lta4h*^*–/–*^ mice (Figure 1A-B), which lack the key enzyme required for synthesizing this mediator. Liquid chromatography-tandem mass spectrometry confirmed the absence of LTB4 in the pellet and supernatant of *Lta4h*^*–/–*^ platelets stimulated with collagen-related peptide, thrombin, or both, whereas arachidonic acid levels remained unaffected. The production of 5(S),12(S)-DiHETE, a stereoisomer of LTB4, was also unchanged, confirming the specificity of Lta4h deletion (Figure 1C; supplemental Figure 1A). Next, we assessed whether platelet-derived LTB4 affects canonical platelet functions. Comparisons of the overall blood parameters and glycoprotein expression on platelets between wild-type (WT) and *Lta4h*^*–/–*^ mice revealed no significant differences (data not shown). The absence of Lta4h did not affect platelet integrin activation (using the conformation-specific JON/A-phycoerythrin. antibody) (supplemental Figure 1B) or platelet degranulation (measured using anti–P-selectin antibody staining) (supplemental Figure 1C). Furthermore, thrombus formation under flow conditions and hemostasis, assessed by tail-bleeding time, were comparable between the groups (supplemental Figure 1D-F). Treatment of WT platelets with the Lta4h inhibitor Lys-006 also did not affect platelet functionality (data not shown). These results suggest that LTB4 is dispensable for canonical platelet functions.

**Figure 1.**
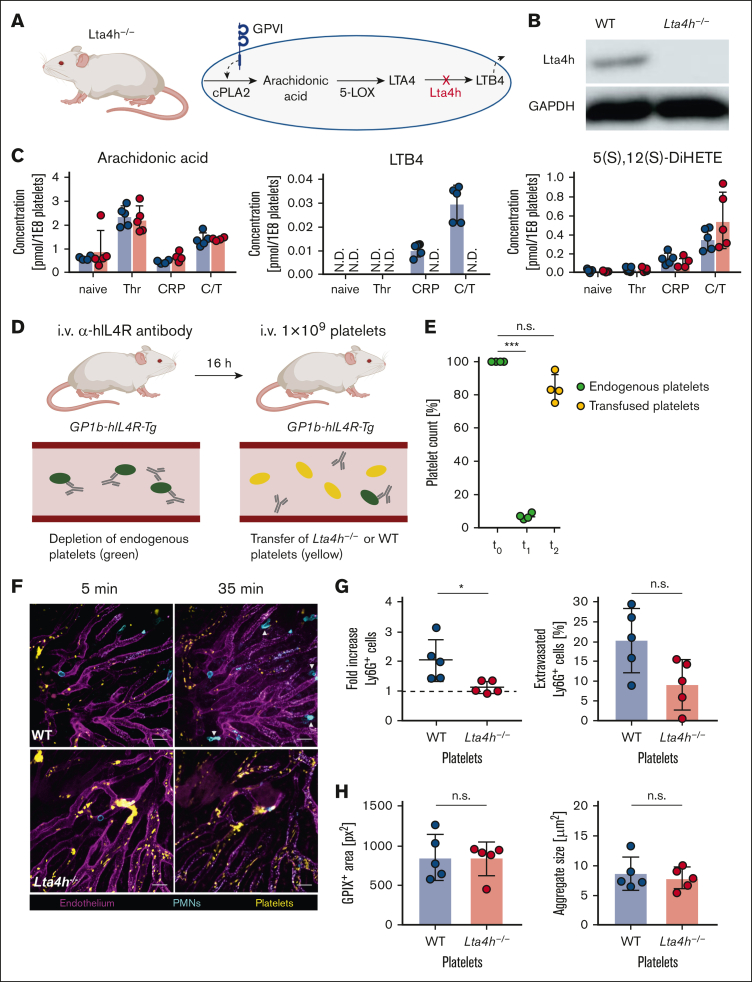
**Platelet-derived LTB4 promotes neutrophil recruitment in acute inflammation.** (A) Schematic representation of LTB4 formation in platelets (figure created using BioRender.com). (B) Western blot analysis of WT and *Lta4h*^*–/–*^ platelet lysates. (C) The concentrations of arachidonic acid, LTB4, and its stereoisomer 5(S),12(S)-DiHETE in the supernatant of WT (blue) and *Lta4h*^*–/–*^ (red) platelets after stimulation with Thr, CRP, or both (C/T) as measured by liquid chromatography-tandem mass spectrometry. (D) Diagram of the adoptive platelet-transfer model in *GP1b-hIL4R-Tg* mice. (E) Platelet counts in *GP1b-hIL4R-Tg* mice at baseline (t0), 16 hours after anti-hIL-4R antibody application (t1), and after platelet transfusion (t2). (F) Representative intravital confocal microscopy images showing neutrophil recruitment (cyan) toward a thermal injury in the liver of *GP1b-hIL4R-Tg* mice transfused with either WT or *Lta4h*^*–/–*^ platelets (yellow). Injury is located in the upper left corner and vessels are shown in magenta. White arrows indicate the extravasated neutrophils. Scale bar: 20 μm. (G) Fold increase (relative to baseline observed 5 minutes after injury induction) of neutrophils per field of view (FOV) (left) and the percentage of extravasated cells 35 minutes after injury induction (right). (H) Total platelet accumulation (left) and mean aggregate size (right) after 35 minutes per FOV. Data points represent the average of 5 FOVs per mouse (n = 5). ∗, *P* < 0.05; ∗∗∗, *P* < 0.001; CRP, collagen-related peptide; C/T, CRP and thrombin; GAPDH, glyceraldehyde-3-phosphate dehydrogenase; h, hours; Thr, thrombin; min, minutes; n.s., nonsignificant; N.D., not detected.

To elucidate the role of platelet-derived LTB4 in thrombo-inflammation, we employed an adoptive platelet-transfer model, because attempts to generate platelet-specific Lta4h-knockout mice using platelet factor 4-cre produced incomplete knockouts (data not shown). We used *GP1b-hIL4R-Tg* mice whose endogenous platelets express the human interleukin-4 (IL-4) receptor ectodomain instead of murine platelet glycoprotein Ibα, thereby enabling selective depletion with an anti-human IL-4 receptor antibody.^10^ After 16 hours, mice were transfused with 10^9^ WT or *Lta4h*^*–/–*^ platelets, which lacked the human IL-4 receptor epitope and thus evaded depletion by residual antibody, thereby enabling replenishment of the physiological platelet counts. Transfusion with WT or *Lta4h*^*–/–*^ platelets ensured that only platelets lacked LTB4 production capacity, whereas other cells, particularly neutrophils, were unaffected (Figure 1D-E). In a thermal hepatic injury model,^11^ we monitored neutrophil accumulation around the injury site via intravital microscopy (Figure 1F). Mice reconstituted with WT platelets showed rapid and significant neutrophil accumulation, which was markedly reduced in mice transfused with *Lta4h*^*–/–*^ platelets (Figure 1G), whereas the total platelet accumulation and mean aggregate size were unchanged (Figure 1H). This underscores the role of platelet-derived LTB4 in promoting neutrophil recruitment during early inflammation. Although not statistically significant, a trend toward reduced neutrophil extravasation was observed in the *Lta4h*^*–/–*^ group (Figure 1G).

Elevated LTB4 levels are found in the bronchoalveolar lavage fluid of patients with asthma and idiopathic pulmonary fibrosis^5^ where neutrophil extravasation into the pulmonary airspace is critical for releasing cytotoxic factors and disrupting the alveolar capillary barrier.^12^ Recently, a role for platelets in this process has been identified based on the reduction in neutrophil adhesion and alveolar infiltration observed in glycoprotein VI (GPVI)-deficient mice, however, the exact mechanism remains unclear.^13^ We used a murine model of lipopolysaccharide (LPS)-induced acute lung injury in *GP1b-hIL4R-Tg* mice transfused with either WT or *Lta4h*^*–/–*^ platelets. Four hours after LPS instillation, bronchoalveolar lavage fluid cell counts were significantly lower in mice that received *Lta4h*^*–/–*^ platelets than in those with WT platelets (Figure 2A). Cytospin differential cell counts showed that most infiltrating leukocytes were neutrophils (86.8 ± 3.2% WT; 85.8 ± 0.2% *Lta4h*^*–/–*^; Figure 2B), highlighting the key role of platelet-derived LTB4 in neutrophil extravasation during acute lung inflammation. Interestingly, collagen-related peptide, which activates platelets via GPVI but not thrombin, induced LTB4 synthesis (Figure 1C), implicating GPVI-mediated LTB4 generation as a critical component in GPVI-dependent thrombo-inflammatory pathways.^13^^,^^14^

**Figure 2.**
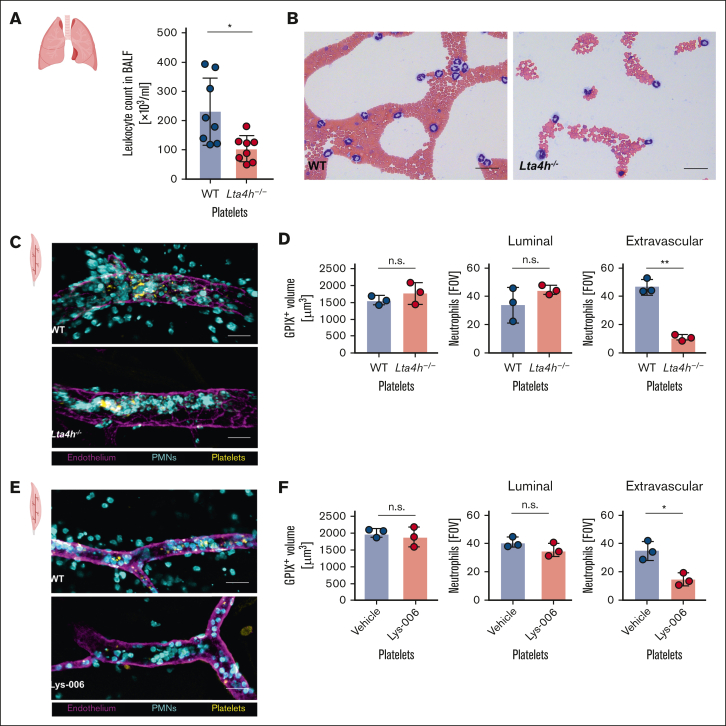
**Platelet-derived LTB4 drives neutrophil extravasation in inflammation.** (A) The total cell counts in bronchoalveolar lavage fluid (BALF) of *GP1b-hIL4R-Tg* mice transfused with either WT or *Lta4h*^*–/–*^ platelets in an LPS-induced acute lung injury model. Each data point represents an individual mouse (n = 8). (B) Representative images of Pappenheim-stained BALF cytospin preparations. Scale bar = 40 μm. (C,E) Representative whole-mount confocal microscopy images of the cremaster muscle from *GP1b-hIL4R-Tg* mice transfused with either WT or *Lta4h*^*–/–*^ platelets (C) or WT platelets treated with Lys-006 or vehicle (E) after an intrascrotal injection of LPS (1 μg/mouse). Images were taken 3 hours after injection and show neutrophils (cyan), platelets (yellow), and postcapillary venules (magenta). Scale bar: 40 μm. (D,F) Quantification of platelet accumulation per FOV (left) and the luminal (middle) and extravasated neutrophils (right), representing neutrophil transmigration. Each data point represents the average number of neutrophils from 10 FOVs per mouse (n = 3). ∗, *P* < 0.05; ∗∗, *P* < 0.01; n.s., nonsignificant; PMN, polymorphonuclear neutrophils.

To further investigate neutrophil extravasation, we used an LPS-mediated inflammation model in the cremaster muscle. Although the accumulation of platelets and luminal neutrophils was similar in both groups, extravasation was significantly reduced in *GP1b-hIL4R-Tg* mice transfused with *Lta4h*^*–/–*^ platelets (Figure 2C-D). This aligns with Subramanian et al who highlighted the LTB4-BLT1 axis in neutrophil arrest and extravasation, suggesting that neutrophil-derived LTB4 acts via auto- and paracrine signaling to redistribute nonmuscle myosin IIa and integrin β2.^5^ Interestingly, *Alox5*^*−/−*^ neutrophils, which cannot generate LTB4, showed normal extravasation when transfused into *BLT1*^*–/–*^ mice, suggesting the presence of LTB4 produced by resident cells.^5^ Our findings extend this by identifying platelets as a key source of LTB4 that promotes neutrophil extravasation.

There have been efforts to target the LTB4-BLT1 axis pharmacologically with 5-LOX inhibitors and LTA4H-specific inhibitors. To test whether platelet LTB4 contributes to the effects of LTA4H inhibitors in mice, we pretreated WT platelets with the LTA4H inhibitor Lys-006, a highly specific and long-lasting inhibitor,^6^ and transfused them into platelet-depleted *GP1b-hIL4R-Tg* mice. As with *Lta4h*^*–/–*^ platelets, Lys-006-treated platelets significantly reduced neutrophil extravasation after LPS administration when compared with vehicle-treated WT platelets without affecting platelet accumulation (Figure 2E-F), suggesting that the effects of LTA4H inhibitors seen in preclinical rodent studies may, in part, stem from their impact on platelet-derived LTB4.

Our findings position platelet-derived LTB4 as a potential therapeutic target in thrombo-inflammatory diseases in that it contributes to neutrophil recruitment and extravasation without affecting platelet functions or hemostasis. Although previous studies proposed that platelets enhance neutrophil LTB4 production by supplying substrates like arachidonic acid,^15^ our data show that the absence of platelet-derived LTB4 directly impairs neutrophil recruitment and extravasation independent of substrate availability because the release of arachidonic acid remained unchanged (Figure 1C).

Overall, our results reveal a previously unrecognized mechanism through which platelets drive inflammatory responses and identified platelet-derived LTB4 as a key mediator of neutrophil recruitment and extravasation across various inflammatory settings.

**Conflict-of-interest disclosure:** The authors declare no competing financial interests.

## Supplementary Material

Supplemental Methods, Figure, and References
