# Supplementary material for: Neutrophil recruitment depends on platelet-derived leukotriene B4
Source: Blood Adv. 2025 Mar 10;9(9):2226–30. doi: 10.1182/bloodadvances.2024014947 (PMC12083915; doi:10.1182/bloodadvances.2024014947)
Supplement: Supplemental Methods, Figure, and References [file BLOODA_ADV-2024-014947-mmc1.pdf]

Platelet-derived leukotriene B4 promotes neutrophil recruitment – Online Material

Short title: Platelet-LTB4 promotes neutrophil recruitment

Christian Hackenbroch, M.Sc.[1,2], Tugce Cimen, M.Sc.[1], Carina Gross, PhD[1,2], Stefanie Rubenzucker M.Sc.[3,4], Philipp Burkard, PhD[1,2], Niklas Groß[1,2], Robert Ahrends, PhD[3], Tamara Girbl, PhD[1], David Stegner, PhD[1,2]

[1] Julius-Maximilians University of Würzburg, Rudolf Virchow Center for Integrative and Translational Bioimaging, Würzburg, Germany

[2] University Hospital Würzburg, Institute of Experimental Biomedicine I, Würzburg, Germany

[3] Department of Analytical Chemistry, University of Vienna, Vienna, Austria

[4] Vienna Doctoral School in Chemistry, University of Vienna, Vienna, Austria

Corresponding authors:

David Stegner, PhD and Tamara Girbl, PhD

Julius-Maximilians-Universität Würzburg, Rudolf Virchow Center for Integrative and Translational Bioimaging and Institute of Experimental Biomedicine, University Hospital Würzburg; Josef-Schneider-Straße 2, 97080 Würzburg, Germany

E-mail addresses: david.stegner@uni-wuerzburg.de or tamara.girbl@uni-wuerzburg.de

Material and Methods

Animals

All animal experiments were approved by the district government of Lower Franconia. Experiments were performed using 8- to 14-week old mice. Lta4h-deficient (*Lta4h*$^{-/-}$) mice [1] and *GP1b-hIL4R-Tg* mice, expressing the chimeric human IL-4 receptor ectodomain on the human GPIbα stalk,[2] were maintained in a specific pathogen-free environment and fed a standard diet ad libitum.

Western Blot

Wildtype and *Lta4h*$^{-/-}$ mice were anesthetized with isoflurane and bled into 300 µl heparin (20 U/ml in TBS, pH 7.3, Ratiopharm). The blood was centrifuged twice at 300*g* for 6 min to obtain PRP. Platelets were pelleted by centrifugation at 800*g* for 5 min and washed twice with Tyrode's buffer (134 mM NaCl, 0.34 mM Na_2HPO_4, 2.9 mM KCl, 12 mM $NaHCO_3$, 5 mM HEPES, 5 mM glucose, 0.35% BSA, pH 7.4) containing 0.02 U/ml apyrase (Sigma-Aldrich) and 0.1 µg/ml PGI_2 (Sigma-Aldrich). Platelets were rested for 30 min at 37°C before being mixed with an equal volume of reducing 2 x loading dye and boiled for 5 min at 95°C. Samples were separated by SDS-PAGE, blotted onto polyvinylidene difluoride membranes, blocked using 10% skim milk powder to prevent nonspecific binding and incubated with a rabbit anti-LTA4H antibody (#STJ24431, St.John´s Laboratory) and a mouse anti-GAPDH antibody (sc-137179, Santa Cruz).

LC-MS/MS oxylipin analysis

Freshly isolated wildtype or *Lta4h*$^{-/-}$ platelets (10^8) were stimulated with either 0.01 U/ml thrombin, 1 µg/ml collagen-related peptide (CRP) or both for 5 min. After centrifugation at 640 *g*, pellets and supernatants were separated and shock-frozen in liquid nitrogen. All sample processing steps were carried out on ice with ice-cold solvents. 225 µL MeOH and 10 µL of an internal standard mix containing deuterated oxylipins were added to the platelet pellet or supernatant. After ultrasonication and thorough vortex mixing, 750 µL MTBE and 20 µL HAc were added. Samples were incubated at 4 °C and 650 rpm for 1 h. Subsequently, 188 µL H_2O were added and the samples were centrifuged at 10,000 *g* and 4 °C for 10 min. The upper organic phase was recovered and dried under a nitrogen stream.

The dried lipid extract was re-suspended in 50 µL eluent A (containing 25 nM CUDA as a system standard) for further MS analysis. To precipitate the protein, 903 µL MeOH were added to the lower phase and the samples were stored at -80 °C for 3 h. Protein pellets were collected after centrifugation at 19,000 *g* for 30 min at 4 °C and resuspended in lysis buffer (1 % SDS, 150 mM NaCl, 50 mM Tris, pH 7.8). Protein concentrations were quantified using a BCA Protein Assay Kit (Thermo Fisher Scientific).

LC-MS/MS analysis were performed on a QTrap 6500+ mass spectrometer (AB Sciex) coupled to a Vanquish Flex UHPLC system (Thermo Scientific). The ESI source settings for the negative mode are as follows: ion spray voltage -4.5 kV, temperature

525 °C, curtain gas 20, ion source gas 1 30, ion source gas 2 55, collision gas medium. Declustering potential, collision energy and collision cell exit potential were optimized individually for each monitored oxylipin. The LC method consists of a 20-minute gradient run on an Ascentis Express C18 column (150 mm × 2.1 mm, 2.7 µm, Supelco), fitted with a guard cartridge (50 mm × 2.1 mm, 2.7 µm, Supelco) and a Vanquish MP35N passive pre-heater. The column compartment and autosampler are kept at 30 °C and 8 °C, respectively. Gradient elution is carried out at 0.4 mL/min with eluent A consisting of ACN/H_2O (3:7, v/v) and eluent B consisting of IPA/ACN (1:1, v/v), both containing 0.1% FA. The linear gradient is as follows: 0–1 min 0% B, 1–13 min increase to 100% B, hold at 100% B for 13–16 min, return to 0% B at 16.1 min, and hold for 3.9 min for re-equilibration. The injection volume is 5 µL, and the injector needle is automatically washed with IPA/ACN 9/1 (v/v) + 0.1% FA before and after each injection. Peak integration and data analysis was carried out using Skyline 22.2.0.315 and KNIME.

Basal blood parameters

Blood parameters were measured in EDTA-blood using a scil Vet abc Plus+ hematology analyzer (scil animal care company GmbH).

Glycoprotein Expression

Blood (50 µL) was withdrawn from the retro-orbital plexus from mice under isoflurane anesthesia and diluted in 300 µL heparin [20 U/mL]. 650 µL Tyrode's buffer without Ca2+ were added to the samples (1:20 dilution). 50 µL of the diluted blood were incubated with fluorophore-conjugated antibodies directed against GPIb, GPVI, GPV, GPIX, α2, α5, αIIbβ3, β3, CD9, Clec2 and CD84 (all in-house generated) and subsequently analyzed via flow cytometry (FACSCelesta, BD Biosciences).

Platelet activation

Blood (50 µL) was withdrawn from the retro-orbital plexus of mice under isoflurane anesthesia, diluted in 300 µL heparin [20 U/mL] and washed twice (5 min, 736 *g*) with 1 mL of Ca^{2+}-free Tyrode's buffer. Afterwards, the washed blood was resuspended in 750 µL Ca^{2+}-containing Tyrode's buffer. Washed blood was incubated with fluorophore-conjugated antibodies directed against activated integrin αIIbβ3 (JON/A^{PE}, in house generated) and against P-selectin (WUG 1.9^{FITC}, in-house generated). Samples were activated with CRP, Rhodocytin (Rhod), ADP, U46619 and thrombin. The reaction was stopped after an incubation of 8 min at 37°C and 8 min at room temperature by addition of 500 µL PBS and platelet activation was analyzed via flow cytometry (FACSCelesta, BD Biosciences).

Flow Adhesion Assay

Glass coverslips were coated with collagen type I (70 µg/ml) and blocked for 1 h with 1% BSA in PBS. Mice were bled up to 1 mL in 300 µL heparin [20 U/mL], the blood was diluted 3:1 with Ca^{2+}-containing Tyrode's buffer, incubated with a DyLight-488-conjugated anti-GPIX antibody [0.2 µg/mL] for 5 min at 37°C and perfused over the collagen for up to 4 min at a shear rate of 1,000/s. Images were captured every 30 s on a DMI6000 B fluorescence microscope (Leica Microsystems) with a 63x oil immersion objective.

Afterwards the collagen-coated surface was washed for 4 min with Ca^{2+}-containing Tyrode's buffer min at a shear rate of 1,000/s and representative images were taken. Surface coverage was analyzed in Fiji.

Tail-bleeding assay

Mice were anesthetized by intraperitoneal injection of midazolam, medetomidine and fentanyl (5, 0.5, and 0.05 mg/kg body weight, respectively) and 1 mm of the tail tip was removed using a scalpel. Tail bleeding was monitored by absorbing blood on filter paper every 20 s without direct contact until cessation or 20 min.

Lys-006 pre-treatment

Isolated WT platelets were incubated with 3 µM Lys-006 (Cay33706, Cayman chemicals) or vehicle control (DMSO) for 30 min at 37°C. Subsequently, platelets were washed twice to remove excess inhibitor and used for experiments.

Platelet-transfer

GP1b-hIL4R-Tg mice were anesthetized with isoflurane before platelets were depleted via the i.v. injection of an anti-hIL4R antibody (2.5 µg/g body weight, MAB230, R&D Systems) [3]. 16 h later, mice were anesthetized using isoflurane and transfused with 1×10^9 platelets isolated from either WT or *Lta4h*$^{-/-}$ mice resuspended in 150 µl Tyrode's buffer.

Hepatic hot-needle injury

For intravital microscopy mice were anesthetized with in an intraperitoneal injection of midazolam, medetomidine and fentanyl (5, 0.5, and 0.05 mg/kg body weight, respectively). Mice were injected i.v. with fluorescently labelled antibodies against neutrophils (anti-Ly6G^{AF647}, clone RB6, in-house generated), platelets (anti-GPIX^{AF488}, in-house generated[4,5]), and endothelial cells (anti-CD105^{AF546}, clone MJ7/19, in-house generated). The operational field was shaved and cleaned before a midline incision. The vessels in the skin and peritoneum were cauterized. The medial liver lobe was exposed, and a cauterizer tip was used to induce a thermal hepatic injury (~300 µm^2)[6]. Intravital microscopy was performed using a TCS-SP8 confocal laser scanning

microscope (Leica Microsystems) with a 25× objective. The microscope was equipped with a Cube Unit to allow a temperature-controlled environment for the mice. Images were acquired with a digital zoom of 2.5 and a resolution of 1024 x 1024 pixels. 20 µm z-stacks were acquired for 5 fields of view (FOV) around the injury every 5 min for 30 min. Z-stacks were processed by Fiji (maximum projection, median filter with pixel size 1) and Ly6G$^+$ neutrophils were counted manually in a blinded fashion.

LPS-induced inflammation of the cremaster muscle

Mice were anesthetized with isoflurane before 1 µg of LPS (O111:B4 Sigma-Aldrich dissolved in sterile PBS) was injected intrascrotally. After a 3-hour incubation period, mice were euthanized, and cremaster muscles were exteriorized for whole-mount immunofluorescence staining. The cremaster muscles were fixed in 4% paraformaldehyde (158127, Sigma-Aldrich) for 1 hour at 4°C and then permeabilized and blocked in PBS containing 0.5% Triton X-100 (9036195, Sigma-Aldrich) and 25% fetal bovine serum (10270106, Gibco) for 4 hours at room temperature. The tissues were incubated overnight at 4°C with unlabeled CD31/PECAM antibody (polyclonal, R&D Systems), Cy3-anti actin α-smooth muscle antibody (clone 1A4, Sigma-Aldrich), anti-GPIX^{AF488} (in-house generated[4,5]) and anti-S100A9/MRP14^{AF647} in 200 µl PBS containing 10% fetal bovine serum. After washing, the cremaster muscles were incubated with Alexa Plus^{405}-IgG (H+L) secondary antibody (A48259, Invitrogen) in 200 µl PBS containing 10% fetal bovine serum for 3 hours at room temperature. After washing, the cremaster muscles were whole mounted for imaging using a TCS-SP8 confocal laser scanning microscope (Leica Microsystems) with a 20x water objective. Ten 35 µm z-stacks of postcapillary venules, as characterized by a diameter of 30-60 micrometers, were acquired per mouse and MRP14$^+$ neutrophils were manually quantified using the Imaris software.

LPS-induced lung injury

Mice, under 2% isoflurane anesthesia, were intranasally instilled with LPS (*Escherichia coli*; O111:B4 serotype; Sigma Aldrich) at a dose of 5 mg/kg body weight (bw) to induce an acute alveolar inflammation. After 4 h, when neutrophil recruitment in the lung vasculature peaked, mice were anesthetized by intraperitoneal injection of ketamine (100 mg/kg bw) and xylazine (16 mg/kg bw). Mice were euthanized via exsanguination, and a bronchoalveolar lavage was performed through 3 washes, each with 600 µL PBS. The bronchoalveolar lavage fluid (BALF) was pooled, total cell counts were determined with a Neubauer improved hemocytometer.

Statistics

Statistical analyses were performed with GraphPad Prism version 9.0.0. Data distribution was analyzed using the Shapiro-Wilk test, and differences were statistically analyzed using a two-tailed Wilcoxon rank-sum test or the Fisher exact test (Fig. 2E). P values ≤ .05 were considered significant (*$P < 0.05$, **$P < 0.01$, ***$P < 0.001$). Data are expressed as mean ± SD.

Supplemental figures

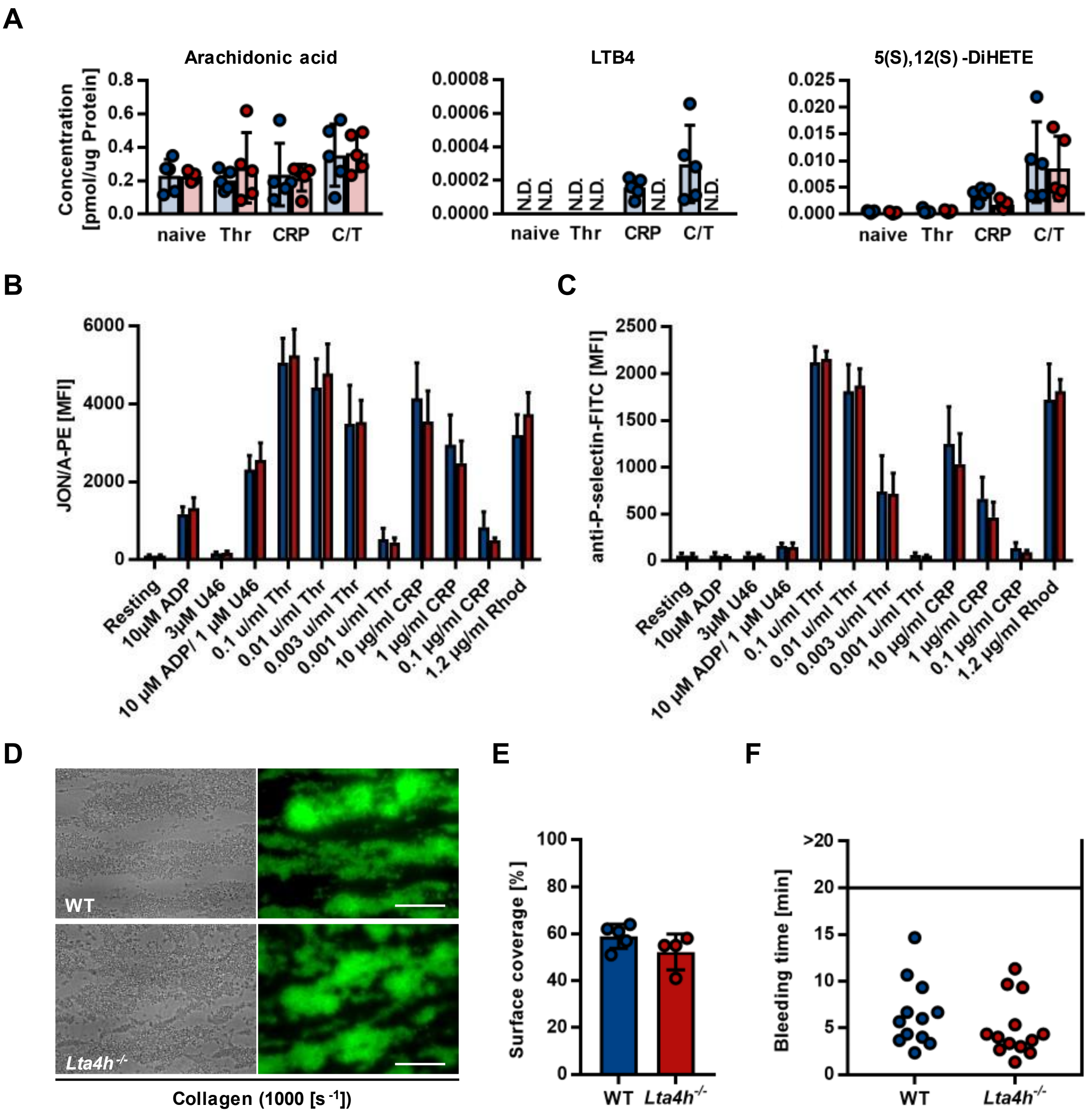

Figure S1: LTB4 is dispensable for canonical platelet function. (**A**) Concentrations of arachidonic acid, LTB4, and its stereoisomer 5(S),12(S)-DiHETE in pellets of WT (blue) and *Lta4h*$^{-/-}$ (red) platelets after stimulation with thrombin (Thr), collagen-related peptide (CRP), or both (C/T), measured by LC-MS/MS. (**B,C**) Integrin αIIbβ3 activation (**B**) and P-selectin exposure (surrogate of platelet degranulation (**C**) of WT (blue) and *Lta4h*$^{-/-}$ (red) platelets measured by flow cytometry. (n=5). (**D**) Representative brightfield and fluorescent images of aggregates formed under flow on collagen from WT and *Lta4h*$^{-/-}$ mice. Scale bar: 20 µm. (**E**) Quantification of surface coverage. Each data point represents an individual mouse (n=8). (**F**) Comparison of tail-bleeding times between WT and *Lta4h*$^{-/-}$ mice. Each data point represents one mouse.

Supplemental references

1. Byrum RS, Goulet JL, Snouwaert JN, Griffiths RJ, Koller BH. Determination of the contribution of cysteinyl leukotrienes and leukotriene B4 in acute inflammatory responses using 5-lipoxygenase- and leukotriene A4 hydrolase-deficient mice. *J Immunol*. 1999;163(12):6810-6819.
2. Kanaji T, Russell S, Ware J. Amelioration of the macrothrombocytopenia associated with the murine Bernard-Soulier syndrome. *Blood*. 2002;100(6):2102-2107.
3. Boulaftali Y, Hess PR, Getz TM, et al. Platelet ITAM signaling is critical for vascular integrity in inflammation. *J Clin Invest*. 2013;123(2):908-916.
4. Stegner D, vanEeuwijk JMM, Angay O, et al. Thrombopoiesis is spatially regulated by the bone marrow vasculature. *Nat Commun*. 2017;8(1):127.
5. Stegner D, Göb V, Krenzlin V, et al. Foudroyant cerebral venous (sinus) thrombosis triggered through CLEC-2 and GPIIb/IIIa dependent platelet activation. *Nat Cardiovasc Res*. 2022;1(2):132-141.
6. Wang J, Hossain M, Thanabalasuriar A, Gunzer M, Meininger C, Kubes P. Visualizing the function and fate of neutrophils in sterile injury and repair. *Science*. 2017;358(6359):111-116.
